# Far-field optical imaging with subdiffraction resolution enabled by nonlinear saturation absorption

**DOI:** 10.1038/srep18845

**Published:** 2016-01-04

**Authors:** Chenliang Ding, Jingsong Wei

**Affiliations:** 1 Shanghai Institute of Optics and Fine Mechanics, Chinese Academy of Sciences, Shanghai 201800, People’s Republic of China; 2 University of Chinese Academy of Sciences, Beijing 100049, People’s Republic of China

## Abstract

The resolution of far-field optical imaging is required to improve beyond the Abbe limit to the subdiffraction or even the nanoscale. In this work, inspired by scanning electronic microscopy (SEM) imaging, in which carbon (or Au) thin films are usually required to be coated on the sample surface before imaging to remove the charging effect while imaging by electrons. We propose a saturation-absorption-induced far-field super-resolution optical imaging method (SAI-SRIM). In the SAI-SRIM, the carbon (or Au) layers in SEM imaging are replaced by nonlinear-saturation-absorption (NSA) thin films, which are directly coated onto the sample surfaces using advanced thin film deposition techniques. The surface fluctuant morphologies are replicated to the NSA thin films, accordingly. The coated sample surfaces are then imaged using conventional laser scanning microscopy. Consequently, the imaging resolution is greatly improved, and subdiffraction-resolved optical images are obtained theoretically and experimentally. The SAI-SRIM provides an effective and easy way to achieve far-field super-resolution optical imaging for sample surfaces with geometric fluctuant morphology characteristics.

With the development of material sciences, microelectronic industry, and opto-electric technoligy, the optical imaging resolution is required to reduce to the subwavelength scale or even the nanoscale. In general, the resolution of light imaging is restricted by the Abbe limit, which is defined as 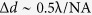 for laser scanning microscopy (LSM), where *λ* and NA are the wavelength of the light source and numerical aperture of the lens, respectively. There are two ways to improve the resolution. One is to reduce *λ*, which has been decreased to 405 nm thus far. However, a wavelength shorter than 405 nm may easily cause strong linear absorption for most samples. The other is to increase the NA of the lens; thus far, the NA has been increased to ~1.49, which is close to the refractive index of cover glass.

However, subwavelength or nanoscale imaging remains difficult because of the Abbe limit. In order to solve this problem, many types of methods and techniques^1^,^2^, such as the near-field methods^3^,^4^,^5^,^6^ and far-field fluorescence-labeling techniques, have been proposed^7^,^8^,^9^,^10^,^11^,^12^,^13^,^14^. The near-field methods meet some difficulties in near-field spacing controlling and low imaging speed, and the far-field fluorescence-labeling techniques are usually applied in the life and biological sciences. Recently, for the fields of material sciences and microelectronics etc, some good ideas and methods are also proposed, such as nonlinear photothermal microscopy^15^, pump-probe subdiffraction-limited imaging^16^,^17^,^18^,^19^,^20^, high-resolution imaging with immersion microscale spherical lenses^21^,^22^, metalens imaging^23^,^24^, and single-objective selective-plane illumination microscopy^25^.

In the present study, inspired by scanning electronic microscopy (SEM) imaging, we propose a saturation-absorption-induced far-field optical super-resolution imaging method (SAI-SRIM). It is well known that in the microscopic structure observation of nonconductive sample surfaces using SEM, a carbon (or Au) thin film is usually required to deposit on the sample surface before imaging to remove the charging effect while imaging by electrons. Our SAI-SRIM is similar to the SEM imaging, the carbon (or Au) layers are replaced by nonlinear-saturation-absorption (NSA) thin films, which are directly deposited onto the sample surfaces using advanced thin film deposition techniques, such as atomic layer deposition method and magnetron-controlled sputtering technique, where the root mean square (RMS) of surface roughness can be controlled at less than 1–2 nm through optimizing the deposition conditions. Then, the sample surfaces are imaged using conventional LSM. Consequently, the imaging resolution is greatly improved, and subdiffraction-resloved optical images are obtained theoretically and experimentally. The proposed SAI-SRIM is very helpful in subdiffraction-resolved far-field optical imaging for the sample surfaces with geometric fluctuant morphology characteristics.

## Physical Pictures of SAI-SRIM

**Figure 1:**
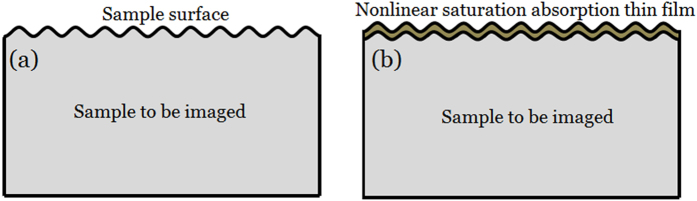
Schematic of the sample structure. (**a**) The sample to be imaged, (**b**) NSA thin film deposited on the sample surface.

Similar to the carbon (or Au) thin films in SEM imaging, in SAI-SRIM, thin films with obvious NSA characteristics are directly deposited on the sample surface. Figure 1a shows the sample to be imaged; the sample surface is not flat and has a microscopic geometric fluctuant morphology. The NSA thin films are directly deposited onto the surface of the sample. The shape of the NSA thin films is modulated by the microscopic geometric fluctuant morphology of the sample surface, as shown in Fig. 1b; that is, the surface morphology of the sample is completely replicated by the NSA thin films.

**Figure 2:**
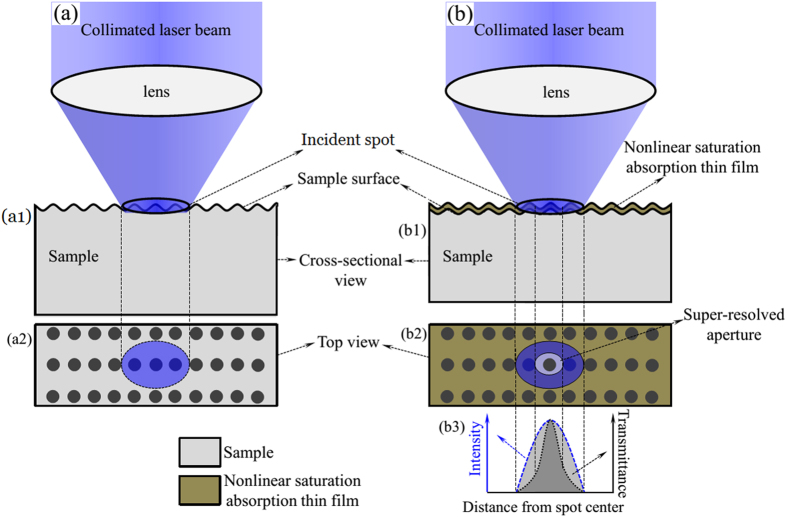
Physical pictures of LSM and SAI-SRIM. (**a**) Conventional LSM, (a1) cross-sectional view, and (a2) top view. (**b**) SAI-SRIM, (b1) cross-sectional view, (b2) top view, and (b3) intensity and transmittance profiles.

The NSA thin films help to improve the resolution beyond the Abbe limit to realize subdiffraction-resolved optical imaging. The basic physical picture is shown in Fig. 2. Figure 2a shows conventional LSM imaging, in which a collimated laser beam passes through a lens and is focused onto the sample surface. The incident spot directly raster-scans the sample surface, imaging its morphology. However, according to Abbe’s theory, two objects cannot be resolved when the distance between them is less than the diffraction limit. Thus, it is not possible to resolve small pits if there are two or more pits within the spot, as shown in Fig. 2a; Fig. 2a(a1) is the cross-sectional view and Fig. 2a(a2) is the top view of conventional LSM imaging.

However, when a thin film with obvious NSA characteristics is deposited on the sample surface, the imaging resolution can be greatly improved, as shown in Fig. 2b. Figure 2b(b1) shows that the morphology of the sample surface is completely replicated by the NSA thin film. The collimated laser beam passes through the lens and is focused onto the NSA thin film. The NSA effect is induced at the incident spot, and the absorption coefficient is expressed as


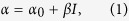


where *α*_0_ and *β* are linear and nonlinear absorption coefficients, respectively, and *I* is the spot intensity. For NSA thin films, *β *< 0, which implies that a higher laser intensity results in a smaller absorption coefficient and, accordingly, a larger transmittance^26^. Therefore, for irradiation with an incident spot having a Gaussian intensity profile, the central transmittance is maximal, and the transmittance decreases in the radial direction of the incident spot, as shown in Fig. 2b(b3). Thus, a super-resolved aperture is produced at the center of the incident spot, as shown in Fig. 2b(b2). Figure 2b(b2) is the top view of Fig. 2b(b1). Only one pit exists within the super-resolved aperture, and other pits within the incident spot are masked by the NSA thin film owing to strong light absorption effect. The light beam passes through the super-resolved aperture and a below-diffraction-limited super-resolved spot is accordingly formed. Thus, pits with central distance among them less than the Abbe resolution limit can be detected, and subdiffraction-resolved far-field optical imaging is achieved using the super-resolved spot/aperture to raster-scan the sample surface.

## Results and Analysis

### NSA characteristics of Sb thin films

**Figure 3:**
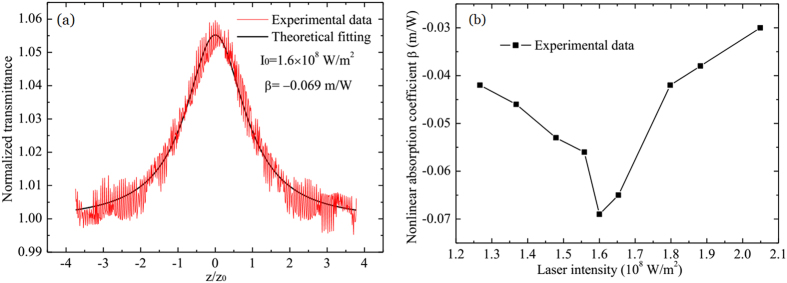
Z-scan measurement results for the Sb thin film. (**a**) Typical z-scan measurement results at 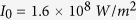. (**b**) Dependence of *β* on laser intensity.

In SAI-SRIM, the NSA thin films are critical, and some basic requirements should be met. One is strong linear absorption, and the other is the strong nonlinear saturation absorption effect. The Sb thin films have a large linear absorption coefficient of 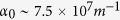 27 and excellent optical switching properties, and they have been successfully applied to super-resolution optical recording and readout^28^,^29^,^30^,^31^. Thus, Sb thin layers are chosen as the NSA thin films. Figure 3a shows a typical open-aperture z-scan measurement result for irradiation with a laser of 405 nm wavelength and light intensity 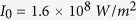. Clearly, the Sb thin film shows the NSA effect. The melting point of Sb materials is only about 630 °C, and the molten-ablation damage occurs easily when the high laser power is used in the experiment. To ensure the effectiveness of the experimental results, the z-scan measurements were repeated many times, and the same results were obtained, indicating that the Sb thin films are not damaged in the process of the z-scan measurement. The nonlinear absorption coefficient is fitted using the equations in ref. [32] and 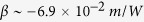, which is basically consistent with the data reported in ref. [27]; the small difference may have originated from the sample preparation process.

Figure 3b shows the dependence of *β* on the laser intensity. *β* first decreases from 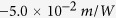 to 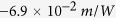 when the laser intensity is increased from 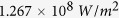 to 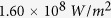, and *β* subsequently increases to 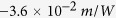 when the laser intensity is reduced to 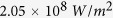. Compared with other materials, the *β* values of Sb thin films are very large, which may be the result of the thermally induced weakening of resonant bonding. As was shown in ref. [27], there is a thermally-induced nonlinear absorption coefficient *β*_*t*_. The *β*_*t*_ is influenced from two aspects, one is the Sb thin film itself, such as surface electron states and defects. The other is from heat diffusion because the the heat diffusion has an effect on the temperature profile. The influence from heat diffusion is reflected by the laser power. The different heat diffusion rate occurs due to the temperature difference between the central and edge regions of spot. When the light intensity is not very high, the temperature difference between centre and edge increases with the light intensity increasing, 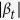 may increases, too. However, when the light intensity is high but not exceed the molten-ablation damage threshold, the temperature difference between centre and edge decreases with the light intensity decreasing, so 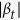 may decreases, too. The detained reasons that the nonlinear absorption coefficency shows power dependent changes need be further analyzed and studied in future work.

### Frequency response function of SAI-SRIM

For far-field optical imaging, the frequency response function (or modulation transfer function) is usually used to characterize the imaging resolution of the optical system. Thus, to understand better how the NSA effect contributes to super-resolution optical imaging, the frequency response function *R*(*f*) is introduced to analyze the imaging resolution of SAI-SRIM. As shown in Fig. 2b, with considerations for the spatial frequency, the sample surface can be considered a grating with discrete orders, and the signal modulation can be determined from the interference of the direct beam in the far field with the similar shifted first-order beams. If the *R*(*f*) of SAI-SRIM exceeds the cutoff frequency of the optical imaging system, the desired level of resolution is expected to be achieved. In the calculations, only the detected region in the far field has to be considered; thus, the frequency response function *R*(*f*) is given by^33^


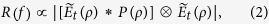


where 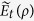 is the Fourier transform of *E*_*t*_(*r*), which is the distribution of the electric field transmitted from the NSA thin films. P(*ρ*) is the Fourier transform of the aperture function *P*(*r*) of the lens, the symbol 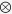 denotes the convolution operation, and


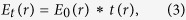


where *E*_0_(*r*) is the electric-field distribution of the incident spot and *t*(*r*) denotes the amplitude transmission of the NSA thin films. *E*_0_(*r*) can be expressed as


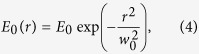


where *E*_0_ is the on-axis electric field amplitude and *w*_0_ is the radius of the incident spot. The value of *t*(*r*) can be roughly calculated using the Lambert–Beer formula. One has


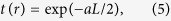


where *L* is the thickness of the NSA thin films. By substituting formula (1) into formula (5), one obtains


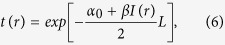


where 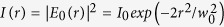. 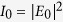 is the on-axis light intensity. *P*(*ρ*) can be expressed as:


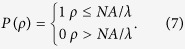


**Figure 4:**
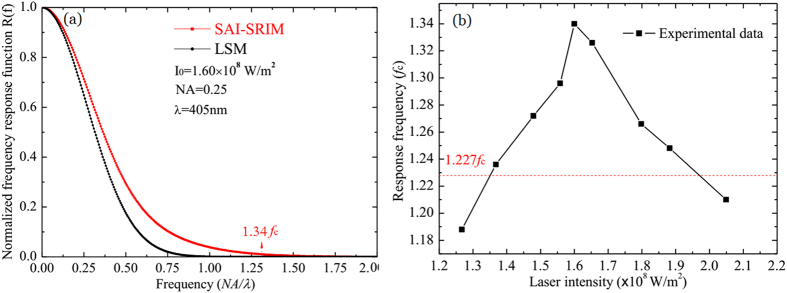
Calculated curves of the frequency response function. (**a**) Comparison of normalized frequency response functions of conventional LSM and SAI-SRIM. (**b**) Dependence of the response frequency of SAI-SRIM on laser intensity.

As an example, we consider that the Sb layer is used as the NSA thin film and that the frequency response function of SAI-SRIM is calculated according to formulas (2)–(7). The results are shown in Fig. 4. The laser wavelength is 405 nm, and the NA of the lens is set to 0.25. Figure 4a shows a comparison of the *R*(*f*) of SAI-SRIM and a conventional LSM imaging system, where the light intensity of the incident spot is 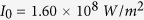. The *f*_*c*_ is defined as the cutoff frequency for conventional LSM. For SAI-SRIM, a normalized *R*(*f*) signal of less than 1% is considered undetectable and accordingly defined as the cutoff frequency. The black curve is the *R*(*f*) of conventional LSM, and the red curve is the *R*(*f*) of SAI-SRIM with the Sb thin film used as the NSA layer; the Sb thin film is directly deposited onto the sample surface. A comparison of the red and black curves indicates that the cutoff frequency of SAI-SRIM is increased to 1.34*f*_*c*_. That is, SAI-SRIM can theoretically realize subdiffraction-resolved far-field optical imaging.

Figure 4b shows the dependence of the response frequency of SAI-SRIM on laser intensity, which is opposite to the dependence of *β* on laser intensity. The response frequency increases from 1.188*f*_*c*_ to 1.34*f*_*c*_ when the laser intensity increases from 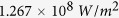 to 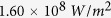; then, the response frequency decreases to 1.21*f*_*c*_ at a laser intensity of 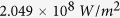.

### Subdiffraction-resolved optical imaging with SAI-SRIM

**Figure 5:**
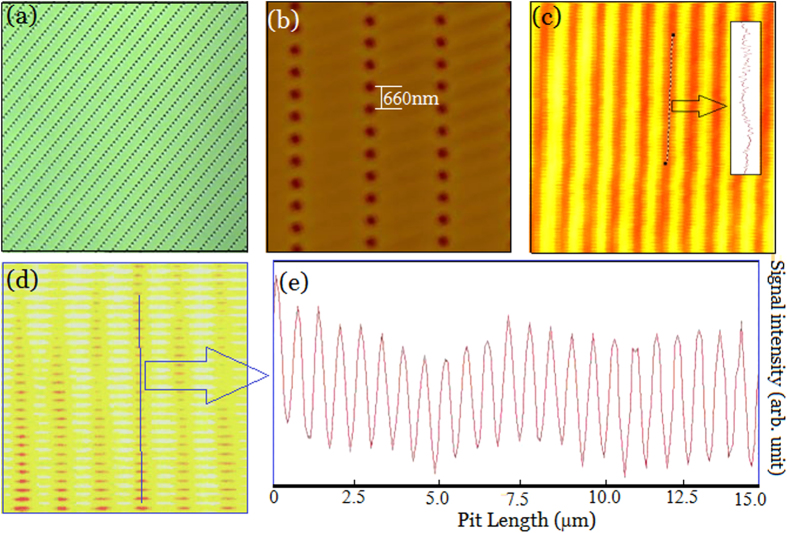
Super-resolution optical imaging of sample surfaces. (**a**) Optical microscopy imaging. (**b**) AFM images (the central distance between adjacent pits is 660 nm). (**c**) LSM imaging (the inset is the cross-sectional profile along the marked line). (**d**) SAI-SRIM imaging with Sb thin films. (**e**) Cross-sectional profile along the marked line in (**d**).

Experiments were conducted to verify SAI-SRIM. An LSM was first established. The light source was a GaN semiconductor laser device with a wavelength of 405 nm, and the NA of the lens was 0.25. The theoretical resolution limit for LSM is 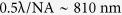. Practically, the resolution limit should be slightly greater than 810 nm because the system cannot be perfect. Polycarbonate substrates with regular pit arrays prefabricated on sample surface were used as samples to verify the SAI-SRIM imaging. The pit arrays were first observed using wide-field optical microscopy with *NA *= 1.40. As shown in Fig. 5a, it was confirmed that regular pit arrays exist on the sample surface. Furthermore, the central distance between two adjacent pits was measured using atomic force microscopy (AFM), as shown in Fig. 5b. The central distance between two adjacent pits was measured as 660 nm, which is less than the resolution limit of LSM. That is, it is impossible to resolve the pit arrays using LSM with *λ *= 405 nm and *NA *= 0.25. Figure 5c indicates that no pit arrays can be observed, and the inset, which shows the cross-sectional profile along the marked-dashed line, also clearly indicates that the pit arrays are unresolvable.

The Sb thin films were directly deposited onto the sample surface using magnetron-controlled sputtering method, which was typical SAI-SRIM procedure. The sample is again imaged using the same LSM at a laser intensity 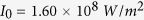. Figure 5d presents the experimental results. One can see that although the shape of pits may be slightly different from that of the original pits, the adjacent pits are clearly resolved, as also observed from the cross-sectional profile of the pit array image shown in Fig. 5e, in which the pit array is resolved and the signal is very clear. Thus, these results verify that the SAI-SRIM imaging is experimentally realized and that the experimental scheme is feasible.

**Figure 6:**
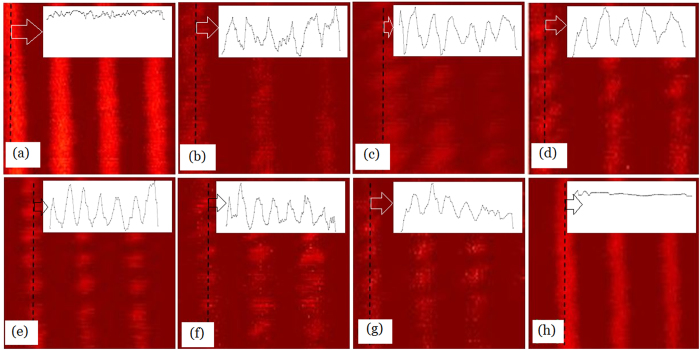
Super-resolution imaging of sample surface under different laser intensities. (**a**) 1.267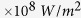. (**b**)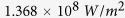. (**c**)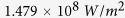. (**d**)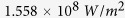. (**e**)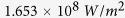. (**f**)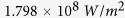. (**g**)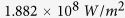. (**h**)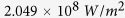.

According to Fig. 4b, the cutoff frequency of SAI-SRIM is dependent on the laser intensity, and the imaging resolution is influenced accordingly. Figure 6 shows the subdiffraction-resolved imaging at different laser intensities, and the insets show the cross-sectional profiles of the pit arrays along the dashed lines. Figure 6a shows the image obtained at 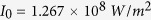; the pits cannot be resolved because the cutoff frequency at 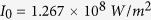 is 1.188*f*_*c*_, which is less than the spatial frequency (1.227*f*_*c*_) of pit arrays, which is marked with red in Fig. 4b. Figure 6b shows the image obtained at 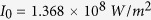; the pits are faintly resolved because the cutoff frequency at 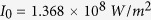 is 1.236*f*_*c*_, which is slightly greater than 1.227*f*_*c*_. One can see from Fig. 6a–e that, with increasing laser intensity, the pit arrays become clearer and resolvable because the cutoff frequency increases with increasing laser intensity. The image is most clearly resolvable at 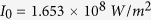, which corresponds to a response cutoff frequency of 1.326*f*_*c*_, which is greater than 1.227*f*_*c*_.

However, on increasing the laser intensity further from 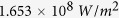 to 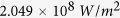, as shown in Fig. 6e–h, the images become increasingly faint and unresolvable because the response cutoff frequency decreases on further increasing the laser intensity, as shown in Fig. 4b. At 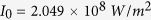, the response cutoff frequency is only 1.21*f*_*c*_, which is less than the spatial frequency (1.227*f*_*c*_) of pit arrays; thus, the pit arrays become completely unresolvable, as shown in Fig. 6h. That is, the images are the clearest at an optimum laser intensity. In our experiments, the optimum laser intensity was 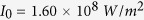. Thus, in real applications, one can use the optimum laser intensity to obtain clear high-resolution images.

Here let us give a rough analysis on the influence of modulation depth on the far-field super-resolution optical imaging. Modulation depth *α*_0_*L* is the product of the linear absorption coefficient *α*_0_ and the thin film thickness *L. α*_0_ usually changes little for given nonlinear absorption thin films, Thus, the modulation depth may mostly be determined by the thin film thickness *L*. As pointed out in ref. [34], the size of super-resolution spot produced by the nonlinear saturation absorption effect decreases with the thin film thickness increasing. However, in real applications, the transmittance of thin film also decreases, accordingly. Thus, the super-resolution performance may enhance, and reach an optimum value, and then decrease with the thin film thickness increasing.

In addition, we also notice that some two-dimensional materials, such as graphene and MoS_2_, have strong nonlinear absorption effects^35^,^36^, and the nonlinear absorption measurements were carried out under femtosecond or picosecond laser pulse. In SAI-SRIM, the imaging is conducted using 405 nm GaN-diode continous wave laser beam. The laser power stability is about 1–2%. The femtosecond and picosecond laser pulses are usually unstable compared with the diode-based continous wave laser beam. Additionally, the nonlinear absorption coefficient of two-dimensional materials is far smaller than the Sb thin films. The two-dimensional materials may be not good for SAI-SRIM. However, we will measure and study the nonlinear absorption properties of two-dimensional materials at 405 nm laser wavelength, and try to introduce the two-dimensional materials into SAI-SRIM in next work.

In summary, inspired by SEM imaging, we proposed SAI-SRIM for overcoming the Abbe resolution limit to realize subdiffraction-resolved far-field optical imaging. The physical picture indicates that the formation of a super-resolved aperture is essential for achieving subdiffraction-resolved images in SAI-SRIM. The theoretical calculation of the frequency response function indicates that the response cutoff frequency is obviously increased with SAI-SRIM. We performed subdiffraction-resolved optical imaging, in which a thin Sb layer was used as an NSA thin film. LSM with 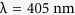 and NA = 0.25 was used as the imaging system, the theoretical resolution limit of which is 810 nm. We used a sample with pit arrays, and the central distance between two adjacent pits was 660 nm, which is lower than the resolution limit of 810 nm. With the SAI-SRIM imaging, the pit arrays were clearly imaged and experimentally resolvable. Thus, the SAI-SRIM was verified both theoretically and experimentally. In the SAI-SRIM, and the imaging process is easy to operate because of just coating a thin Sb layer onto the sample surface, and the strict conditions or complex imaging systems are not needed. Thus, the proposed method is a simple and very effective for subdiffraction-resolved far-field optical imaging for the sample surfaces with geometric fluctuant morphology characteristics.

## Methods

### Preparation of Sb thin film

A Sb thin film with a thickness of approximately 100 nm was deposited on a substrate by using a direct-current magnetron-controlling sputtering method at room temperature. The sputtering power was 30 W, the background pressure was approximately 3 × 10^−4^ Pa, and the sputtering pressure was 0.75–0.8 Pa in an Ar environment. The sputtering speed and time were 0.5 nm/s and 200 s, respectively.

### Measurement of nonlinear absorption

The nonlinear absorption properties of Sb thin films were measured using the open-aperture z-scan method. A semiconductor laser with a wavelength of 405 nm was chosen as the irradiation source because the imaging was conducted with a laser device of 405 nm wavelength. The experimental setup is described in ref. [32].

### Preparation of sample to be imaged

Optical imaging was conducted using LSM with a laser wavelength of 405 nm and NA of 0.25. In the SAI-SRIM imaging, the Sb thin films were directly deposited onto the sample surface to be imaged using the direct-current magnetron-controling sputtering method. The pit arrays on the sample surfaces were prepared using laser lithography method, and the central distance between adjacent pits was 660 nm.

## Additional Information

**How to cite this article**: Ding, C. and Wei, J. Far-field optical imaging with subdiffraction resolution enabled by nonlinear saturation absorption. *Sci. Rep.* **6**, 18845; doi: 10.1038/srep18845 (2016).
